# A decade of decline: Grant funding for researchers with disabilities 2008 to 2018

**DOI:** 10.1371/journal.pone.0228686

**Published:** 2020-03-03

**Authors:** Bonnielin K. Swenor, Beatriz Munoz, Lisa M. Meeks

**Affiliations:** 1 The Wilmer Eye Institute, Johns Hopkins University School of Medicine, Baltimore, Maryland, United States of America; 2 Department of Epidemiology, Johns Hopkins Bloomberg School of Public Health, Baltimore, Maryland, United States of America; 3 Department of Family Medicine, The University of Michigan Medical School, Ann Arbor, Michigan, United States of America; Università degli Studi di Perugia, ITALY

## Abstract

Recent data highlights an imbalance in research grant success among groups underrepresented within the biomedical workforce, including racial/ethnic minorities and women. However, there is no data on grant success for researchers with disabilities.

For these analyses, aggregate data on self-reported disability status for National Institute on Health (NIH) research grant applicants and awardees was obtained from 2008 to 2018, including disability category: mobility/orthopedic, hearing, visual disabilities, and other disabilities. The percentage of applications and awards, as well as grant success rates (% of applicants receiving awards), by Principal Investigators (PIs) disability status were calculated. Data was desegregated, and logistic models determined trend of applicants reporting disability over time. The percentage of NIH grant applicants with PIs reporting a disability significantly declined from 1.9% in 2008, to 1.2% in 2018 (p<0.001). Data on grant awardees was similar, 1.9% of awards in 2008, declining to 1.2% in 2018 (p<0.001) had PIs reporting a disability. Across all years, the percentage of applications and awards with PIs reporting visual disabilities was lower than the percentage reporting mobility/orthopedic, or hearing disabilities (16.5%, 34.2%, and 37.8% in 2008, respectively). Overall grant success rates differed by disability status (27.2% for those reporting disability vs 29.7% in those reporting no disability, p<0.001). The lowest overall grant success rate was among PIs reporting unknown disability status or who withheld this status (18.6%). These results underscore the underrepresentation of researchers with disabilities among grant applicants and awardees, and indicate lower grant success rates among PIs reporting disabilities.

## Introduction

Calls for greater diversity in science and medicine have been strengthened by recent data highlighting an imbalance in research grant success among groups underrepresented within the biomedical workforce, including women [1–4] and racial/ethnic minorities [5–7]. Federal funding agencies, including the National Institutes of Health (NIH) and National Science Foundation (NSF) maintain a commitment to attracting and retaining candidates from underrepresented groups [8,9]. Specifically, the NIH “encourages institutions to diversify their student and faculty populations to enhance the participation of individuals from groups identified as underrepresented in the biomedical, clinical, behavioral, and social sciences”[10]. The NIH definition of underrepresented backgrounds includes individuals with disabilities, which is defined as “those with a physical or mental impairment that substantially limits one or more major life activities” [10].

Despite the stated commitment to the inclusion of persons with disabilities, this population remains largely omitted from biomedical workforce diversification efforts. This omission is evident by the limited reporting and surveillance of the representation of persons with disabilities in the biomedical workforce [11]. For example, recent NIH reports aimed on workforce diversity have not included data on employees with disabilities[5–7]. Data from the 2017 NSF’s report “*Women*, *Minorities*, *and Persons with Disabilities in Science and Engineering”* is among the only sources of data on the representation of persons with disabilities in science. This report indicates that researchers with disabilities are underrepresented in science, as only 10% of employed scientists and engineers report having a disability[12], and is in comparison to the 26% of American adults reporting a disability[13]. However, the data used for this NSF report does not include researchers with medical degrees, which limits the generalizability of these estimates.

For persons with disabilities, the barriers to a successful research career are multifactorial and mirror those experienced by other underrepresented groups including social and cultural bias, lack of concordant mentoring, and structural barriers to engaging in the research process [14]. Social and cultural bias may include misperceptions and stigma about the abilities of people with disabilities, exclusion from the social and scholarly activities that support career development like academic conferences, invited talks, and collaborative scholarship. Stigma and bias threaten disability disclosure in academia, which holds implications for career success including reduced potential for mentorship and sponsorship, known catalysts for research career success [15], and limits opportunities to manage structural barriers ranging from physical space inaccessibility to obtaining needed adaptive and assistive technologies.

Grant funding is an important indicator of research career success [16]. Recent data highlights an imbalance in research grant success among groups underrepresented within the biomedical workforce, including racial/ethnic minorities and women, amplifying the need to consider the career and research implications of being a minority in these settings [1–5], yet there are no reports on grant success for researchers with disabilities.

Documented accounts of disability bias during grant review exist [17], yet there is limited research in this area. While not apparent, by reviewing a grant application, it is possible that bias during grant review occurs when a researcher’s disability status is known by peers reviewing the grant. Despite the efforts underway to promote the inclusion of people with disabilities in the biomedical workforce, and research examining barriers to research grant success for other underrepresented groups in science and medicine, it remains unknown if the research career success for these individuals differs by disability status leaving a gap in our understanding of an important metric in career success for this group [18,19].

Here, we present data on the number and percentage of National Institutes of Health (NIH) grant applicants and awardees. We hypothesize that researchers reporting disabilities will have decreased research grant success as compared to those not reporting disabilities.

## Materials and methods

The NIH collects data on grant applicants’ disability status via self-report under the demographics section within the eRACommons portal. The Principal Investigator’s (PI) disability status is determined by their response to the question “Do you have a disability?” (response options are “yes”, “no”, or “do not wish to provide”, as describe below. This section includes the statement “This information is used only for aggregate statistical reporting.” A unique digital identifier can be used to connect researchers and their contributions to science, including grant applications and awards, providing insight into the NIH grant success rates by PI disability status.

Data used for these analyses was obtained via a Freedom of Information Act (FOIA) request. Aggregate data was provided, which included the annual number and percentage of NIH grant applications and awards from PIs classified into one of three disability categories within the eRACommons (Table 1): (1) *Reported* *Disability*: applications or awards where the PI responded “yes” to the question “Do you have a disability?” *Disability type* is reported by selecting all categories that apply: mobility/orthopedic, hearing, visual, or other; (2) *No Disability*, defined as applications or awards where the PI indicated “no” to the question “Do you have a disability?”; and (3) *Unknown/Withheld Disability Status*, which is a combined category calculated as the sum of applications or awards where the query “Do you have a disability?” was left blank by the PI (defined as “unknown disability status”) or where the PI selected “Do not wish to provide” in response to the “Do you have a disability?” question (defined as “withheld disability status”). The data for this category was provided from the NIH as the composite of both the unknown and withheld groups (as shown in Table 1).

**Table 1:** Number and percentage of National Institute of Health Grant Applications and Awardees by Principal Investigator Disability Status: 2008 to 2018.

**Fiscal Year**	**NIH Grant Applicants**						
	**# Applicants**	**# Applicants Reporting Disability**	**% Applicants Reporting Disability**	**# Applicants Reporting No Disability**	**% Applicants Reporting No Disability**	# Applicants with Unknown/ Withheld Disability*	% Applicants with Unknown/ Withheld Disability*
2008	40,050	746	1.9%	30,963	77.3%	8,341	20.8%
2009	39,796	731	1.8%	33,210	83.5%	5,855	14.7%
2010	46,042	774	1.7%	39,906	86.7%	5,362	11.6%
2011	48,251	752	1.6%	42,189	87.4%	5,310	11.0%
2012	48,911	731	1.5%	42,518	86.9%	5,662	11.6%
2013	47,506	672	1.4%	40,285	84.8%	6,549	13.8%
2014	48,627	634	1.3%	41,477	85.3%	6,516	13.4%
2015	48,768	596	1.2%	41,811	85.7%	6,361	13.0%
2016	51,234	651	1.3%	43,922	85.7%	6,661	13.0%
2017	51,497	608	1.2%	44,119	85.7%	6,770	13.1%
2018	52,124	617	1.2%	44,599	85.6%	6,908	13.3%
**NIH Grant Awardees**							
**Fiscal Year**	**Total # Awardees**	**# Awardees Reporting Disability**	**% Awardees Reporting Disability**	**# Awardees Reporting No Disability**	**% Awardees Reporting No Disability**	# Awardees with Unknown/ Withheld Disability*	% Awardees with Unknown/ Withheld Disability*
2008	12,409	235	1.9%	10,743	86.6%	1,431	11.5%
2009	12,052	213	1.8%	10,646	88.3%	1,193	9.9%
2010	12,817	207	1.6%	11,448	89.3%	1,162	9.1%
2011	12,354	165	1.3%	11,102	89.9%	1,087	8.8%
2012	12,731	189	1.5%	11,510	90.4%	1,032	8.1%
2013	11,773	159	1.4%	10,601	90.0%	1,013	8.6%
2014	13,364	165	1.2%	12,064	90.3%	1,135	8.5%
2015	13,689	153	1.1%	12,409	90.6%	1,127	8.2%
2016	14,811	172	1.2%	13,466	90.9%	1,173	7.9%
2017	14,920	175	1.2%	13,563	90.9%	1,182	7.9%
2018	16,441	191	1.2%	14,741	89.7%	1,509	9.2%

(1) Research grant applicants and awardees are included where research grants are defined as: R, P, M, S, K, U (excluding UC6), DP1,DP2, DP3, DP4, DP5, D42, G12.

(2) Multiple Principal Investigators are included.

(3) Disability data are self-reported based on information reported by the individual in the eRA Commons system. An individual may choose not to report or withhold disability information.

(4) ARRA is excluded.

(5) Only competing applications and awards are included.

(6) Applications withdrawn prior to review are excluded.

* The number of applicants or awardees with Unknown/ Withheld disability is the sum of applicants or awardees without a disability code and those with a withheld disability code of 'W'.

The data provided included disability information for PIs on the following grant mechanisms: R, P, M, S, K, U, DP1, DP2, DP3, DP4, DP5, D42, G12. The resulting aggregate data included information from Multiple Principal Investigators (MPI) grants, and included only competing applications. American Recovery and Reinvestment Act (ARRA) grants, UC6 grants, and applications withdrawn prior to review were excluded from these data. Data were provided at the level of applications and awards and were provided in aggregate, independent of how many grants each applicant/PI submitted or was awarded in any given year.

The percentage of application and awards with PIs reporting disability by category was calculated as the sum of responses for each disability category divided by the total number reporting a disability. Grant success rate was calculated as the percentage of grant applications that received funding.

To examine differences and trends over time, data was desegregated and logistic models were used to determine differences in the proportion of applicants reporting disabilities over time. Trends of the grant success rate over time were not linear and yearly data was combined using a Mantel-Haenzel approach to determine differences in funding rates by disability status; odds ratios and 99% confidence intervals are reported.

Analyses were performed using SAS (SAS Institute, Cary, NC) software.

## Results

**Fig 1:**
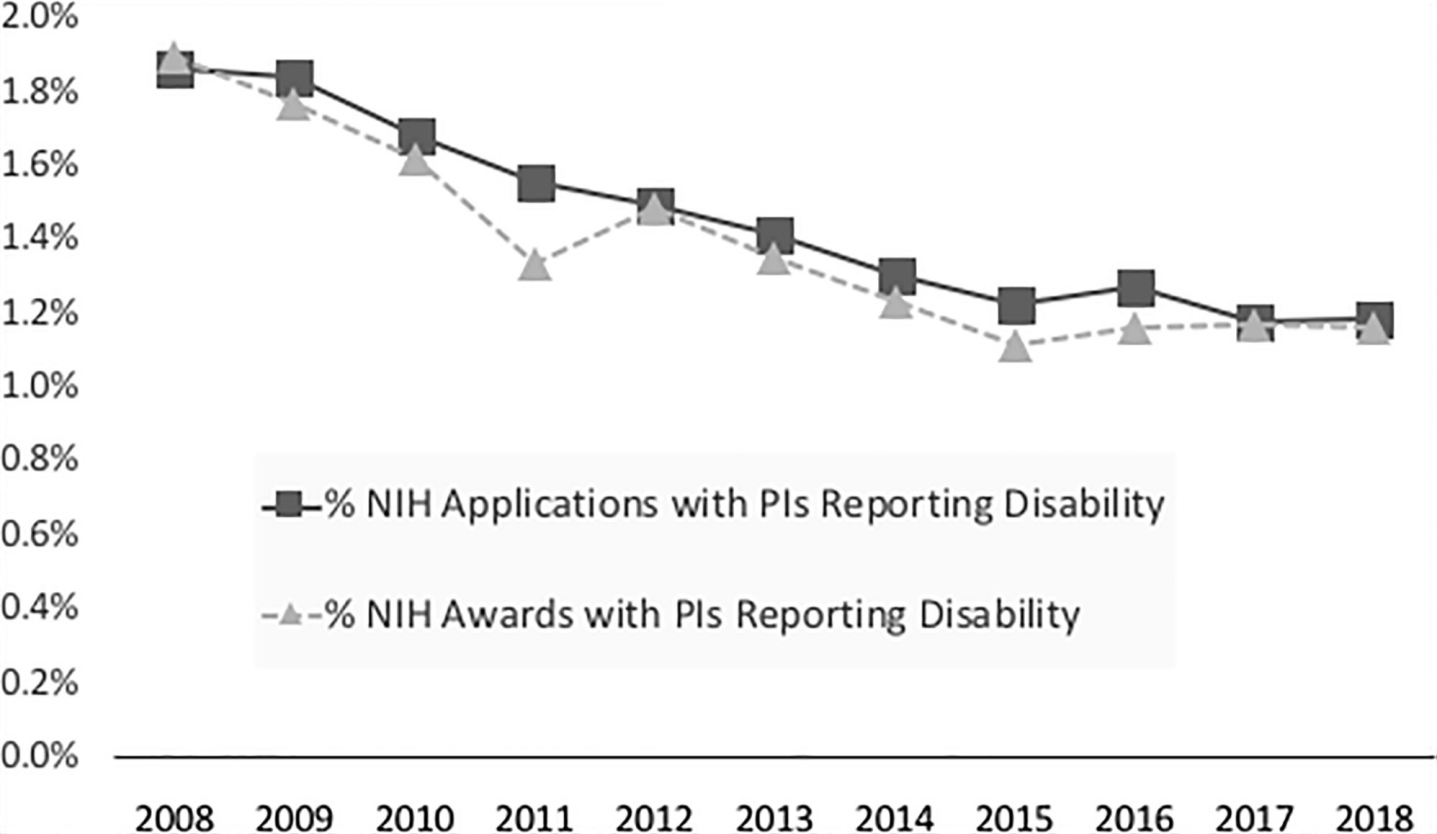
Percentage of National Institute of Health Grant Applications and Awards from Principal Investigators Reporting Disability.

In 2008, 1.9% of NIH grant applications included a PI reporting a disability (Table 1 and Fig 1). This percentage declined over the subsequent decade to 1.2% in 2018 (OR of annual decline = 0.95; 99% CI: 0.94–0.96; p<0.001). Reciprocally, over the same period, the percentage of applications with PIs that did not report a disability increased from 77.3% in 2008 to 85.6% in 2018. Data on grant awardees, mirrored the applicant data. In 2008, 1.9% of grants awarded included PIs reporting a disability (Table 1 and Fig 1) and this percentage declined to 1.2% in 2018 (OR for annual decline = 0.95; 99% CI: 0.93-.97, p <0.001). These data are in contrast to grants awarded to PIs not reporting a disability, which increased from 86.6% in 2008 to 89.7% in 2018. The percentage of NIH applications and awards from PIs reporting unknown disability status or who withheld this status significantly declined from 2008 to 2018 (p<0.001) (Table 1).

**Fig 2:**
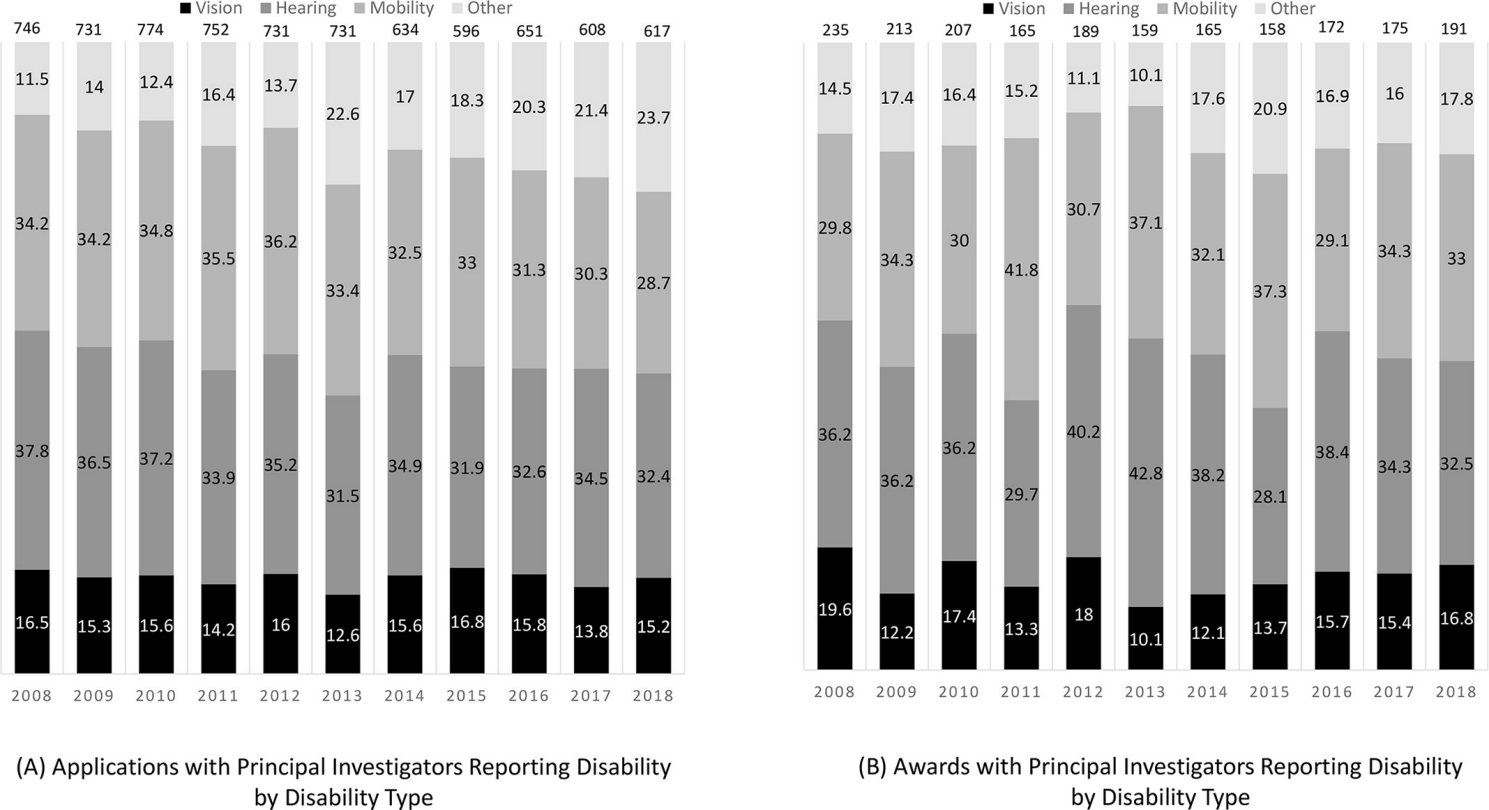
Number and percentage of National Institute of Health (NIH) Grant Applications and Awards with Principal Investigators Reporting Disability by Disability Type: 2008 to 2018. (A) Applications with Principal Investigator Reporting Disability by Disability Type; (B) Awards with Principal Investigator Reporting Disability by Disability Type.

There was no change in the proportion of NIH grant applications and awards from PIs reporting any type of disability (mobility/orthopedic, hearing, visual disabilities, or other) between 2008 and 2018 (p = 0.54 for applicants and p = 0.58 for awardees). (Fig 2). However, when compared across categories, the percentage of PIs with disabilities was lowest for those reporting visual disabilities compared to those reporting mobility/orthopedic, hearing, or other disability categories.

**Fig 3:**
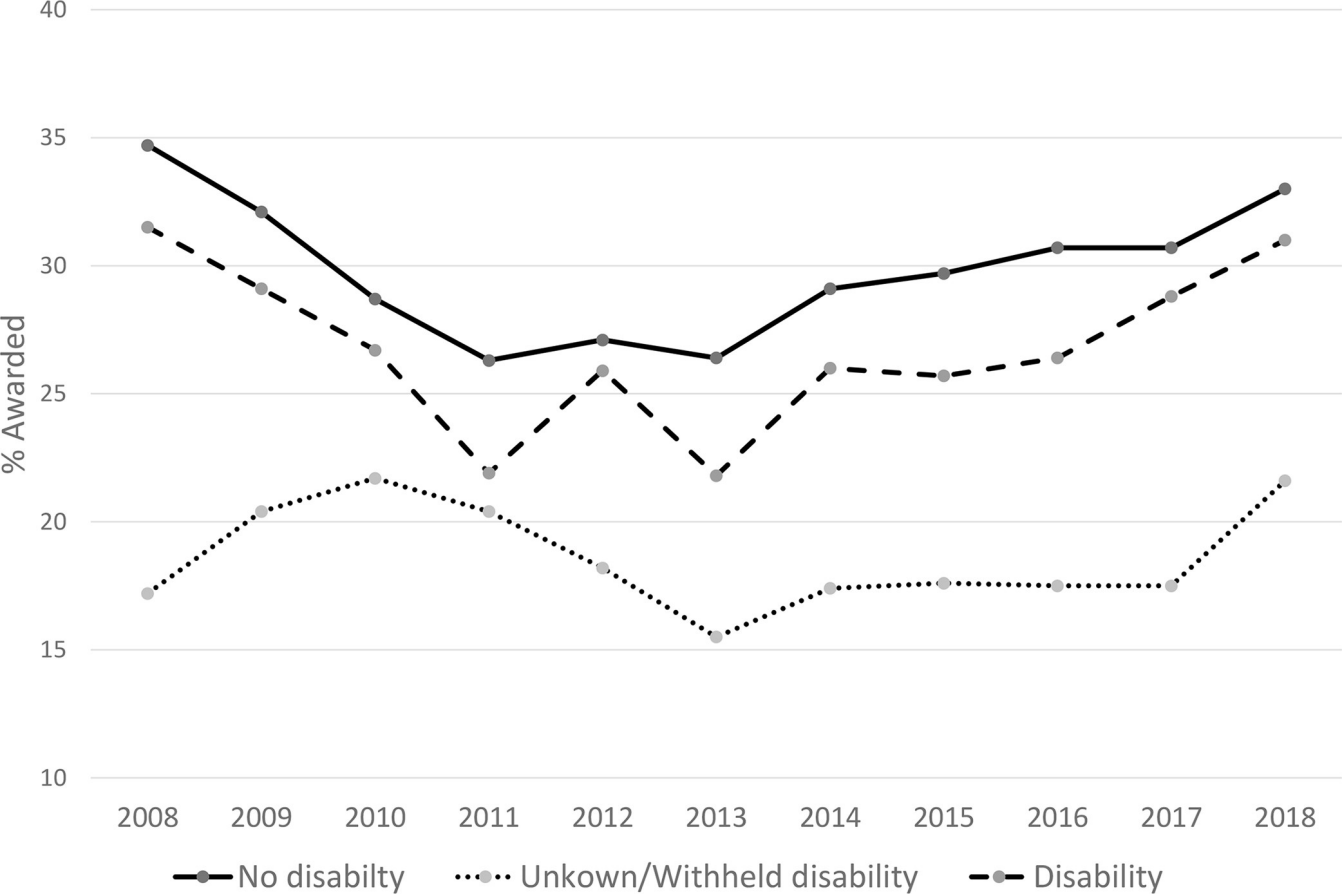
Grant success rate (% of awarded applications) among National Institute of Health Grants by Principal Investigator Disability Status: 2008 to 2018.

Grant success rate was examined using combined data over the entire period (2008 to 2018). Overall funding rates were compared by disability status (PIs reporting disability, PIs reporting unknown or withheld disability status, and PIs reporting no disability). These funding rates were significantly lower for PIs reporting disabilities (27.2%) than for those not reporting disabilities (29.7%) (OR = 0.86; 99% CI: 0.80–0.92; p < 0.001) over this period. The overall funding rate, however, was lowest for applications with PIs reporting unknown disability status or where the PI withheld disability status (compared with those reporting no disability, OR = 0.53 99%CI:0.52–0.55, p<0.001) (Table 2, Fig 3). When funding success rates were examined by disability type (visual, hearing, mobility, or other disability), applications with PIs reporting “other” disability had the lowest success rate (24.6%) among these types (Table 2).

**Table 2:** Grant success rate* among National Institute of Health Grants by year and Principal Investigator Disability Status: 2008 to 2018.

Year	Overall	No Disability (%)	Unknown-withheld (%)	Reported Disability Overall (%)	Disability Category			
					Visual(%)	Hearing(%)	Mobility(%)	Other(%)
2008	31.0	34.7	17.2	31.5	37.4	30.1	27.5	39.5
2009	30.3	32.1	20.4	29.1	23.2	28.8	29.2	36.3
2010	27.8	28.7	21.7	26.7	29.8	26.0	23.0	35.4
2011	25.6	26.3	20.5	21.9	20.6	19.2	25.8	20.3
2012	26.0	27.1	18.2	25.9	29.1	29.6	22.6	21.0
2013	24.8	26.4	15.5	21.8	17.4	29.6	24.2	9.7
2014	27.5	29.1	17.4	26.0	20.2	28.5	25.0	26.9
2015	28.1	29.7	17.7	25.7	21.0	22.6	28.9	29.4
2016	28.9	30.7	17.6	26.4	26.2	31.1	24.5	22.0
2017	28.8	30.7	17.5	28.8	32.1	28.6	32.6	21.5
2018	31.5	33.0	21.8	31.0	34.0	31.0	31.5	23.3
**Overall**	**28.2**	**29.7**	**18.6**	**27.2**	**26.7**	**27.7**	**26.9**	**24.6**

* Grant success rate is calculated as the percentage of grant applications that received funding.

## Discussion

These data indicate that less than 2% of NIH applications and awards include PIs that report a disability, and that the percentage of NIH grant applications and awards from PIs reporting disabilities declined significantly between 2008 and 2018. These results may reflect one or more potential scenarios: (1) that the underrepresentation of biomedical researchers with disability may be worsening over time, (2) that despite increased overall applications to NIH, researchers with disabilities are submitting fewer applications, on average, then researchers without disabilities or (3) that more researchers with disabilities are withholding disability status over time. The percentage of applicants and awards with PIs with unknown or withheld disability status, however, declined from 2008 to 2018 (see below for further discussion), suggesting that the observed decline in the percentage of NIH grant applications and awards from PIs reporting disabilities is not driven by a decline in PI’s willingness to disclose disability. These results mirror similar research that suggest the barriers for female researchers begin in the processes leading up to submitting a grant proposal [20].

When examined by disability category, the percentage of grant applications and awards with PIs reporting visual disabilities was lower than other disability categories. This result suggest that researchers with visual disabilities may have unique barriers that limit applying for and obtaining NIH grants. The aggerate data used for these analyses do not allow us to investigate the reasons for this difference, but do highlight the need for and value of collecting and examining data by disability type to examine and identify barriers to inclusion, as each disability and area of research results in unique barriers rendering a “one-size-fits-all” approach to inclusion antiquated.

Our results further indicate that the grant success rates of PI’s reporting disabilities were significantly lower than PIs not reporting disability over this same period. This result suggests that researchers with disabilities may be disadvantaged in the grant peer review process and mirrors similar research suggesting barriers to grant funding for female and underrepresented researchers. Recent research by Hoppe et al.[21] suggests differences in grant success by African Americans (AA) and black researchers stem from: (1) bias during grant peer review resulting in lower impact scores or because they are less likely to be discussed, although there is conflicting data [22,23], (2) lower percentage of AA/black applicants resubmitting an unfunded application; and (3) differences in research topic choices by race. While the data provided in response to our FOIA do not facilitate investigating the reasons for lower grant success rates among applications from PIs reporting disabilities, access to data similar to that used in the analyses by Hoppe et al. [21] would provide insight into the potential bias and barriers driving the differences in funding success observed in our data.

The percentage of NIH applications and awards from PIs reporting unknown disability status or who withheld reporting this status declined from 2008 to 2018 (Table 1), suggesting increased disability disclosure in eRACommons, yet, this group had the lowest grant success rate over this period (Table 2). This area requires further examination. There are unique barriers to disclosing disability in eRA Commons, including limited categorical options for reporting disability type, with visual, hearing and mobility among the only defined categories. While the option for “other” exists, investigators may be hesitant to self-disclose a category of disability [e.g., psychological, learning, chronic health] that are not distinctly recognized by the NIH and that may carry additional biases or stigma. Our results indicate a need to examine the factors driving the potential increase in the percentage of PIs disclosing disability, in contrast to the low success rates for PIs reporting unknown disability status or who withheld reporting disability status. The reasons PI’s elect unknown disability status or elect to withhold disability status are undetermined within this dataset. PIs may withhold disability status for several reasons, including failure to complete the disability question, fearing that a response would bias their application, category of disability not available, or failure to comprehend the disability question. Similarly, PIs who reported unknown disability status (i.e. selected “Do not wish to provide” in response to the disability questions) may have selected this response due to fear of bias in disclosing or not disclosing disability during the review of their grant application, or not understanding the disability question or absence of categories. Our attempts to obtain aggregate data from the NIH separating applications and awards from PIs withholding disability status from those not reporting disability status (as defined in the Materials and methods section above) was denied.

The limitations of this study include missing information from both PIs who withheld disability responses and those who reported unknown disability status. This potentially influences our reported estimates; however, we cannot determine the direction or magnitude of this impact. Therefore, the results from this group should be interpreted with caution. Additional limitations of these data should also be considered. First, disability status is likely underestimated within this dataset for two reasons: (1) disability is self-reported within eRACommons, and (2) not all categories of disability are queried (*i*.*e*. psychiatric, learning, and chronic health). Data on unknown/withheld disability status suggest a reluctance to disclose disability status within eRACommons. While this likely underreporting results in underestimates of the data presented, the underreporting is, itself, a problem that needs to be addressed. The reluctance to disclose disability status is reflective of the negative stereotypes and stigma that surround disability in the biomedical workforce and academia [13,15,24–27]. Second, only aggregate data was provided in response to our FOIA request. Therefore, we cannot further explore differences in this data by other factors, such as gender, race/ethnicity, age, or by funding mechanism. Third, irrespective of disability status, we cannot determine if PIs submitted grant applications across multiple years, and therefore are unable to account for this potential correlation in our analyses. While aggregate data preserves anonymity, further research, either quantitative or qualitative, is necessary to fully address the reasons for the lower percentages of NIH grant applications and awards with PIs reporting disabilities.

Despite the limitations in this data, our analysis is among the first reports of grant application and awardee data by disability status. These data indicate that individuals with disabilities are underrepresented within the biomedical workforce, may be less likely to apply for research funding, and have lower grant success rates than researchers not reporting disabilities.

Further, our results underscore the urgency to identify and address the barriers to grant funding success for researchers with disabilities. These efforts should consider personal accounts of disability in research and medicine [28,29], and lessons learned from work aimed at including women and groups underrepresented in science and medicine. A broad view of these potential barriers is also needed, which may differ by disability type and setting and consider barriers at each step in the process of developing and submitting a grant application, including difficulty to accessing scientific or grant materials, lack of assistive technology or other types of accommodations, and limited or differential mentorship for this group.

These data also suggest that efforts to realize the NIH’s commitment to the inclusion of biomedical researchers with disabilities will require more specific, focused efforts, programmatic funding and accountability for existing requirements to recruit and retain persons with disabilities. Building on recommendations for other underrepresented groups [30,31] increased representation and inclusion of researchers with disabilities will require evidence-based, milestone-driven changes that address structural-level barriers, include organizational accountability and commitment for disability inclusion, and address challenges to unequal representation in research funding. Therefore, to enact measurable change, the NIH and academic institutions need to move beyond disability policy towards actionable steps that support inclusion by: (1) simultaneously prioritizing recruiting, retaining, and including scientists and clinicians with disabilities in all aspects of career development, including applying for and obtaining grant funding, as well as representation on scientific panels, and study sections; (2) undertaking proportional efforts by disability type, as disparities in NIH funding exists among individuals reporting visual disabilities; and (3) expanding surveillance tools to include psychiatric, learning and chronic disease disabilities, as the absence of additional disability category selections may unintentionally send a message that individuals with these categories of disability are not qualified or welcome as researchers. Refining category type may also aid PI’s in endorsing disability, and electing a category, and could reduce the “unknown” responses in the population, leading to clearer interpretation of that subset of data. Updating the disability data collected in eRACommons is a first step toward collecting more robust data on disability and assess the impact of future policies and efforts to enhance the representation and inclusion of researchers with disabilities with NIH grant funding. These measures are critical to accurate data collection and representation across disability type and may provide important insight on the landscape of disability inclusion in science and medicine.

## Conclusion

Our results indicate that researchers with disabilities are underrepresented among NIH grant funding applicants and have lower grant success rates as compared to researchers not reporting disabilities. Though efforts are underway to enhance the representation of persons with disabilities in the biomedical workforce[15,18,19], these data indicate a need to focus beyond metrics of representation and examine and address barriers to researcher career success, such as grant funding, and to assess the landscape of disability inclusion in research settings. Without a clear understanding of the barriers and trajectories of researchers with disabilities we risk perpetuating a cycle of inaccessibility that negatively impacts entry to and promotion within the biomedical workforce. Moreover, the NIH’s stated commitment to diversity will never be fully realized until the barriers to inclusion of researchers with disabilities are removed.
